# Boron tolerance and accumulation potential of four salt-tolerant plant species

**DOI:** 10.1038/s41598-019-42626-8

**Published:** 2019-04-18

**Authors:** Qian Zhao, Jia Li, Zheng Dai, Chengcang Ma, Hongwen Sun, Chunguang Liu

**Affiliations:** 1 Key Laboratory of Pollution Processes and Environmental Criteria (Ministry of Education), Tianjin Key Laboratory of Environmental Remediation and Pollution Control, Nankai University, Tianjin, 300350 China; 2 College of Life Sciences, Tianjin Normal University, Tianjin, 300387 China

**Keywords:** Environmental biotechnology, Pollution remediation

## Abstract

Boron (B) is an essential element for plants, but excess B is phytotoxic. Since excess B often occurs along with high salinity in the environment, the purposes of the experiments are to screen plants that tolerate both excess B and high salinity for the remediation of B-contaminated saline water or soils. Here we tested the capacities of B tolerance and accumulation of four salt-tolerant plant species, *Tripolium pannonicum*, *Suaeda glauca*, *Iris wilsonii*, and *Puccinellia* *tenuiflora* using hydroponic culture systems, and compared their potential for application in phytoremediation. The maximum B supply concentrations for the survival of *T*. *pannonicum*, *S. glauca*, *I*. *wilsonii*, and *P*. *tenuiflora* are 40, 250, 700, and 300 mg/L, respectively. The maximum B concentrations in the shoot tissue of these plants are 0.45, 2.48, 15.21, and 8.03 mg/g DW, and in the root are 0.23, 0.70, 6.69, and 2.63 mg/g DW, respectively. Our results suggest that *S. glauca*, *I*. *wilsonii*, and *P*. *tenuiflora* are capable of tolerating and accumulating high levels of B, and *I*. *wilsonii* is a most promising candidate for the remediation of B-contaminated sites. This study will provide evidence in support of our future pilot studies (e.g., constructed wetlands) on the phytoremediation of B-contaminated water and soil.

## Introduction

Boron (B) is an essential element for plant growth, but it can be toxic when present in excess^1^. B toxicity in plants has been widely reported in North America, Southern Australia, the Middle East, Western Asia, North Africa, Malaysia, and China^2–4^. Soil B concentrations have been reported to be elevated by anthropogenic activities such as borate mining and processing, glass and ceramic production, as well as the use of B-enriched detergents, herbicides, fertilizers, and irrigation water^2,5–7^. B concentration is usually 0.1–0.5 mg/L in surface freshwaters, but the higher B concentrations are measured in some areas. For example, it has even been documented that B concentration in surface water of Rio Arenales and Loa River (two South American rivers) ranged between 4 and 26 mg/L in areas rich in B-containing soils^8^. High concentrations of boron in water are also very common in China. In an investigation in a B industry area, B concentrations in surface water and groundwater were up to 3.8 mg/L and 140 mg/L, respectively^9^.

Phytoremediation is considered as a green and sustainable technology for the treatments of B-contaminated soil or water, including the restoration of B-mining sites and the purification B-laden effluent^10,11^. When phytoremediation technologies are applied to B-contaminated soils, it is essential to find suitable plant species that can tolerate and/or accumulate high levels of B^12^. Previous studies have recorded some extremely B-tolerant plant species. For example, *Gypsophila sphaerocephala*, *Chrysopogon zizanioides* L., and *Puccinellia distans* were observed to survive up to 227 mg B/kg soil, 750 and 1250 mg B/L hydroponic solution, respectively^13–15^. In addition to B tolerance, some plant species have been reported to accumulate B efficiently. For example, *Phytolacca americana*, *Ambrosia trifida* L., and *Commelina communis* were reported to grow in the soil with 480–550 mg B/kg and obtain tissue B concentrations 2–3 folds greater than the soil^16^. High B-accumulation capacity was also recorded in *Poplar* sp., the leaves of which were observed to accumulate up to 845 mg B/kg dry weight^17^. Apart from terrestrial plants, some aquatic species are reported to be able to accumulate B. For example, a floating aquatic plant, *Lemna gibba*, was reported to accumulate 930 to 1900 mg B/kg dry weight in the tissue^18^.

In the soils of arid and semi-arid regions, excess B often presents simultaneously with excess salt (mostly sodium chloride), which may aggravate B toxicity in plants or affect B tolerance of the plants^12,19^. To remediate the soils with high B and salinity using plants, therefore, it is necessary to screen plant species that tolerate both B and salt. Although the tolerance to B and salt has been demonstrated in some plant species^20–22^, few of which are considered as good accumulators of B. We recently selected four salt-tolerant plant species, *Tripolium pannonicum*, *Suaeda glauca*, *Iris wilsonii*, and *Puccinellia* *tenuiflora*, which are popularly used for the revegetation of saline lands^23–26^. Unfortunately, the capacities of B tolerance and accumulation of these four species are still unknown. In the present work, we cultivated these four plant species in different concentrations of B using hydroponic solutions and then determined plant biomass and tissue B concentrations. We tested B tolerance and accumulation of the four salt-tolerant species and evaluated their potential for the phytoremediation of B-contaminated soils.

## Materials and Methods

### Plant culture

The seedlings of *T*. *pannonicum* and *S. glauca* were collected from Dagang, a coastal area of Tianjin, China (N38°44′43.27″, E117°28′52.77″). The seedlings of *I*. *wilsonii* and the seeds of *P*. *tenuiflora* were obtained from the Tianjin Landscape Institute (N39°5′51.81″, E117°15′55.28″). The seeds of *P*. *tenuiflora* were germinated in sand to obtain seedlings. The seedlings were cultivated in modified half-Hoagland’s solution^15^ in the culture room for 50 days after germination. All seedlings were 7–8 cm in height before B treatment. The seedlings of the four plant species were cultivated in 1-L polyethylene bottles (9 cm in diameter and 18 cm in height), which were filled with 1 L of half-Hoagland’s solution. The bottles were covered with aluminum foil to exclude light, and the solution was aerated continuously. In each bottle, one seedling was grown for *T*. *pannonicum*, *S. glauca*, or *I*. *wilsonii*, and three seedlings were grown for *P*. *tenuiflora*. All the seedlings were acclimated in half-Hoagland’s solution for 2 weeks before treatment. The experiment was conducted in a culture room at 25 ± 2 °C with an irradiance of 72 µmol/m^2^/s supplied with a 12-h photoperiod.

### B treatment

B was added to the half-Hoagland’s solution in the form of boric acid to obtain desired B concentrations. The pH of the solution was adjusted to 6.5. The B supply concentrations ranged from 0.25 to 40 mg/L for *T*. *pannonicum*, from 0.25 to 250 mg/L for *S. glauca*, from 0.25 to 700 mg/L for *I*. *wilsonii*, and from 0.25 to 300 mg/L for *P*. *tenuiflora*. These four ranges of B concentration were established according to preliminary tests for each species. In preliminary tests, the threshold of B concentrations for the four species was determined. And preliminary experimental conditions such as light and pH in the process of the experiment are the same as the formal experiment. The lowest concentration of B (0.25 mg/L) was the “control” treatment (assumed sufficient to prevent B deficiency). Each treatment was triplicated. The solutions were replaced once a week throughout the experiment.

### Plant analysis

After treated with B for 2 weeks, the plants were harvested. The plant samples were rinsed with tap water and then separated into shoot and root. The samples were dried at 75 °C for 24 h and then weighed to determine the dry weight (DW). The dried tissue was ground into powder and then digested using nitric acid/hydrogen peroxide microwave digestion, and the B concentrations in the tissue were quantified using inductively coupled plasma atomic emission spectroscopy (ICP-AES)^15^.

The bioconcentration factor (BCF) of B in plant shoot and root was calculated using the following equation^27^:

1 $$BCF={C}_{p}/{C}_{w}$$

where *C*_*p*_ is the concentration of B in plant tissue (mg/g DW) and *C*_*w*_ is the concentration of B in the culture solution (mg/ml).

The translocation factor (TF) of B was calculated using the following equation^27^:

2 $$TF={C}_{i}/{C}_{r}$$

where *C*_*i*_ is the concentration of B in shoot tissue (mg/g DW) and *C*_*r*_ is the concentration of B in root tissue (mg/g DW).

### Statistics

The data in the figures and tables were reported as the mean ± standard deviation (SD). All the data were analyzed using the program SPSS 17 (IBM Corp., Armonk, NY, USA). One-way ANOVA tests were conducted to determine the influence of B treatment on the dry weight and B concentration in plant tissue of the four species. Prior to ANOVA, the Levene score and significant values of dry weight and B concentration in plant shoot and root were analyzed. The homogeneity of the normal score of variances was verified with Levene’s test. All the tests were conducted with a 95% confidence interval (*α* = 0.05). Duncan’s multiple range tests were used to calculate the significant differences between the means of different treatments at the level of *p* < 0.05. All figures were produced using Origin 8.5 software (OriginLab Corporation, Northampton, MA, USA).

## Results

### Plant growth

**Figure 1:**
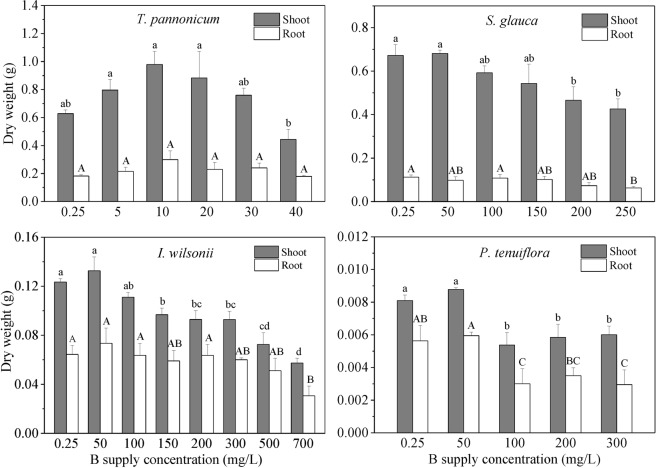
Dry weight of the shoot and root of the four plants grown at different B supply concentrations for 2 weeks. Means with different letters are significantly different (*p* < 0.05). Lowercase and uppercase letters are for the data of shoot and root, respectively.

According to the preliminary tests, the seedlings of *S. glauca*, *I*. *wilsonii*, and *P*. *tenuiflora* were able to survive at 250, 700, and 300 mg B/L, respectively, while *T*. *pannonicum* was unable to survive B concentrations higher than 40 mg/L. With increasing B concentrations, dry weight of the four species increased initially and then decreased progressively (Fig. 1). Shoot DW of *T*. *pannonicum* reached a maximum at 10 mg B/L, and shoot DW of *S. glauca*, *I*. *wilsonii*, and *P*. *tenuiflora* reached a maximum at 50 mg B/L. Significant decreases in shoot DW for *T*. *pannonicum*, *S. glauca*, *I*. *wilsonii*, and *P*. *tenuiflora* were achieved at B supply concentrations of 40, 200, 150, and 100 mg/L, respectively. At these concentrations, compared with the control (0.25 mg B/L), shoot DW of the four species decreased by 29.31%, 30.67%, 21.63%, and 33.70%, respectively. Root biomass showed similar tendencies with shoot biomass but had fewer changes with increasing B supply concentrations. Over the B supply concentrations, root DW of *T*. *pannonicum* showed no significant changes. Root DW of *S. glauca*, *I*. *wilsonii*, and *P*. *tenuiflora* significantly decreased at 250, 700, and 100 mg B/L, respectively.

### B concentrations in plant tissue

**Figure 2:**
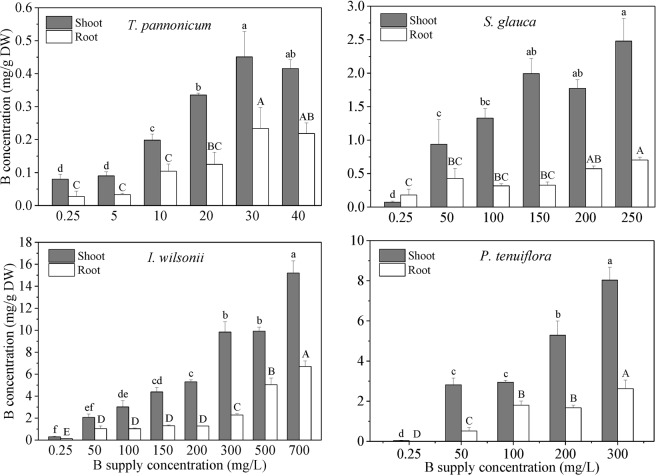
B concentrations of the shoot and root of the four plants grown at different B supply concentrations for 2 weeks. Means with different letters are significantly different (*p* < 0.05). Lowercase and uppercase letters are for the data of shoot and root, respectively.

Shoot B concentrations of the four test species increased with increasing B supply concentrations. Although root B concentration in *P*. *tenuiflora* at B supply concentration of 100 mg/L was higher than that at 200 mg/L, the data of the two groups have no significant difference. But the general trend is that root B concentrations increased with increasing B supply concentrations. B concentrations of the four tested species in shoot were much higher than those in root (Fig. 2).

At B supply concentrations of 30, 250, 700, and 300 mg/L, shoot B concentrations in *T*. *pannonicum*, *S. glauca*, *I*. *wilsonii*, and *P*. *tenuiflora* increased to the maximum values, 0.45, 2.48, 15.21, and 8.03 mg/g DW, respectively. Similarly, at the same B supply concentrations, their maximum B concentrations were recorded in root as 0.23, 0.70, 6.69, and 2.63 mg/g DW, respectively. Compared with the other three species, over the whole range of B concentrations for plant survival, *T*. *pannonicum* showed the lowest capacity of B accumulation. For example, the maximum accumulation of B in the shoot of *I*. *wilsonii* was 15.21 mg/g DW, which was 33.8 times greater than that of *T*. *pannonicum*. And the maximum accumulation of B in the root of *I*. *wilsonii* was 29.09 times greater than that of *T*. *pannonicum*.

**Figure 3:**
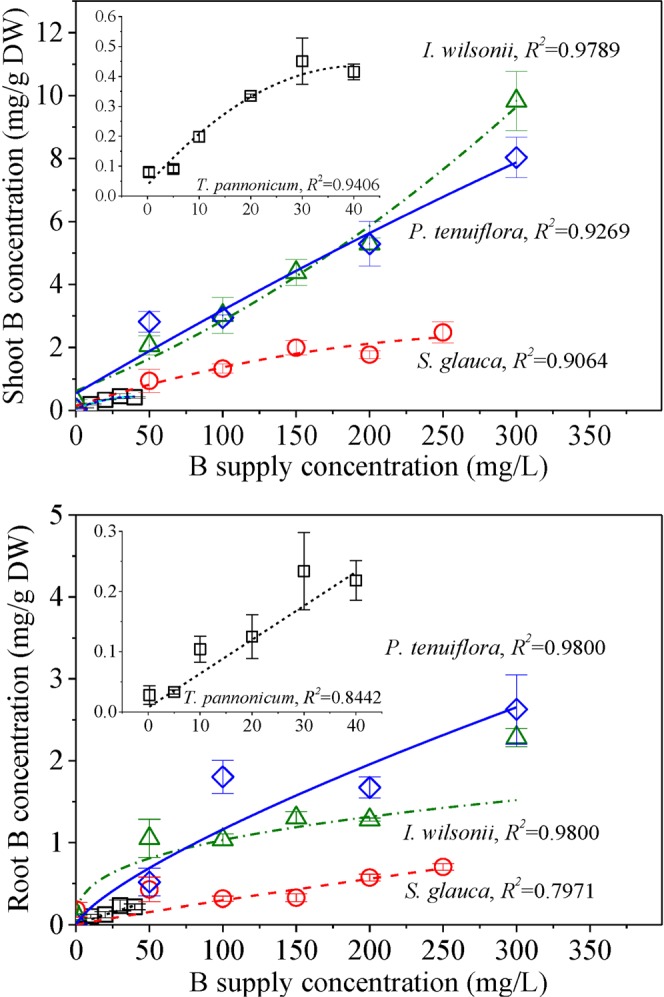
Relationship between B concentration in plant tissue of the four species and in the culture solution. Values shown are the averages, n = 3.

When tissue B concentration of the four plant species is plotted against B supply concentration, there is a high degree of correlation between the two parameters (Fig. 3). Because *S. glauca*, *I*. *wilsonii*, and *P*. *tenuiflora* were all able to grow over the range of 0.25–250 mg B/L, changes in tissue B concentrations between the three species can be compared. Over this range, shoot B concentrations in *I*. *wilsonii* and *P*. *tenuiflora* were similar and greater than those in *S. glauca*. In root, B concentrations of *P*. *tenuiflora* were greater than those in *I*. *wilsonii* and *S. glauca*, especially when B supply concentrations exceeded 100 mg/L.

### B accumulation and translocation in plant

Bioconcentration factor (BCF) and translocation factor (TF) are popularly used for evaluating the potential of plant for the phytoremediation of heavy metals and metalloids^27,28^. The BCF and TF values of B in the four plant species were calculated and shown in Table 1. In the shoot and root of the four species, the BCF values tended to decrease with increasing B supply concentrations. Among the four plant species, *I*. *wilsonii* (BCF in shoot: 19.83–41.3) and *P*. *tenuiflora* (BCF in shoot: 26.44–56.31) showed greater BCF values than *T*. *pannonicum* (BCF in shoot:10.39–19.84) and *S. glauca* (BCF in shoot: 8.78–18.77). For these plant species, BCF values in shoot (mostly exceeded 10) were much greater than those in root. Over the range of 0.25–40 mg B/L, with increasing B supply concentrations, the changes in TF values in *T*. *pannonicum* were not obvious (*p* < 0.05). At relatively higher B supply concentrations, greater TF values were yielded in the test plants except *T*. *pannonicum*. Most TF values in the four plants were greater than 1.0.

**Table 1:** Bioconcentration factor (BCF) and translocation factor (TF) in the four plant species after 2 weeks of exposure to B.

	*T*. *pannonicum*			*S. glauca*			*I*. *wilsonii*			*P*. *tenuiflora*		
	BCF^S*^	BCF^R**^	TF	BCF^S^	BCF^R^	TF	BCF^S^	BCF^R^	TF	BCF^S^	BCF^R^	TF
0.25	160.20^a***^	55.98^a^	2.86^a^	146.68^a^	361.78^a^	0.41^c^	589.63^a^	256.10^a^	2.30^bc^	81.77^a^	26.00^a^	3.14^b^
5	17.99^b^	6.65^a^	2.70^a^									
10	19.84^b^	10.42^a^	1.90^a^									
20	16.76^b^	6.26^a^	2.68^a^									
30	15.03^b^	7.79^a^	1.93^a^									
40	10.39^b^	5.46^a^	1.90^a^									
50				18.77^b^	8.55^a,b^	2.19^b^	41.30^b^	21.02^b^	1.96^b,c^	56.31^b^	10.35^c^	5.44^a^
100				13.28^b^	3.16^b^	4.20^b^	30.16^b^	10.36^b^	2.91^a,b^	29.48^c^	18.02^b^	1.64^c^
150				13.28^b^	2.18^b^	6.10^a^	29.20^b^	8.68^b^	3.36^a,b,c^			
200				8.78^b^	2.86^b^	3.10^b^	26.47^b^	6.40^b^	4.14^a^	26.44^c^	8.37^d^	3.16^b^
250				9.91^b^	2.81^b^	3.53^b^						
300							32.77^b^	7.61^b^	4.31^a^	26.77^c^	8.76^d^	3.06^b^
400												
500							19.83^b^	10.10^b^	1.96^c^			
700							21.73^b^	9.56^b^	2.27^b,c^			

^*^BCF^S^: BCF values in the shoot.

^**^BCF^R^: BCF values in the root.

^***^Means in the same column with different superscript letters are significantly different (*p* < 0.05).

## Discussion

To remediate the soil with excess B and salt, it is important to find candidate plants that are able to tolerate both B and salt and have great capacities of B accumulation. Here we show that three salt-tolerant plant species (*S. glauca*, *I*. *wilsonii*, and *P*. *tenuiflora*) have the capacities of B tolerance and accumulation, especially for *I*. *wilsonii* (tolerates up to 700 mg B/L and accumulates up to 15.21 mg B/g DW in shoot). Our findings have provided potential candidates for the phytoremediation of high-B soils with high salinities.

As a candidate for the remediation of high-B soils, the plant must be able to survive at high B concentrations. We have observed that *T*. *pannonicum* was not able to survive at B concentrations greater than 40 mg/L, while *S. glauca*, *I*. *wilsonii*, and *P*. *tenuiflora* were able to survive 250, 700, and 300 mg B/L, respectively. So far, there are no criteria for the assessment of B tolerance of plants. In general, crop plants are more sensitive to B toxicity than other species. For some tolerant species (e.g., carrot, alfalfa, sugar beet, etc.), 2–4 mg B/L in irrigation water may be harmful concentrations^29^. Some species used for the phytoremediation of B-contaminated soils have exhibited greater B tolerance. For example, *Puccinellia*, a genus of alkali grass, has been demonstrated to tolerate high concentrations of B. Some species of this genus, *P*. *frigid* and *P*. *distans*, have been recorded to survive over 500 mg/L^30^ and 1250 mg B/L^15^ under hydroponic conditions, respectively. In the present study, *P*. *tenuiflora*, a species of *Puccinellia*, was also observed to survive at high B concentrations (up to 300 mg/L).

Besides the survival abilities, the changes in plant biomass also reflect the capacities of plants adapting to B toxicity. Previous studies have demonstrated that B-tolerant species, cultivars, or genotypes exhibit less reduction in biomass or yield than sensitive ones when exposed to B toxicity^31,32^. Our results show that the optimal B concentrations for biomass accumulation were 10 mg/L for *T*. *pannonicum* and 50 mg/L for *S. glauca*, *I*. *wilsonii*, and *P*. *tenuiflora*, rather than 0.25 mg/L (B concentration of half Hoagland’s solution) (Fig. 1). These results are consistent with previous observations in some B-tolerant plant species, which obtained the highest biomass at 2.5 to 50 mg B/L^15,33,34^. The data of the four species in the present study suggest that these species require more B for growth than those provided by the half-Hoagland’s solution. In the present study, when B supply concentrations exceeded 50 mg/L, the biomass of *S. glauca*, *I*. *wilsonii*, and *P*. *tenuiflora* all decreased. The significant drop of shoot biomass for *S. glauca*, *I*. *wilsonii*, and *P*. *tenuiflora* was observed at B concentrations of 200, 150, and 100 mg B/L, respectively, which were quite different with the survival concentrations of B for the three species (250, 700, and 300 mg/L, respectively). These results suggest that the plant species surviving at higher B concentrations may not maintain greater capacities for biomass accumulation at high B concentrations.

A mechanism of B tolerance for plant is to restrict B transfer from root to shoot which restrict the accumulation of B in shoot^13,15,35^. In the present study, tissue B concentrations of *S. glauca*, *I*. *wilsonii*, and *P*. *tenuiflora* were observed to increase with increasing B supply. Compared with B supply concentrations of 50 mg/L, there was no significant increase in shoot B concentrations of *S. glauca*, *I*. *wilsonii*, and *P*. *tenuiflora* until B supply concentrations reached 150, 150, and 200 mg/L, respectively (Fig. 2). These results indicate that the three species are all able to restrict B accumulation in shoot in response to increasing B supply. Over the range of B supply concentrations of 50 to 200 mg/L, *I*. *wilsonii* and *P*. *tenuiflora* obtained higher shoot B concentrations than *S. glauca*, indicating less B was accumulated in *S. glauca* (Fig. 3). Since B concentrations in the root of *S. glauca* were also lower than those in the root of *I*. *wilsonii* and *P*. *tenuiflora*, *S. glauca* is likely to have the ability to restrict B uptake or B efflux from the root.

As is known, greater bioconcentration factor (BCF) and translocation factor (TF) indicate the higher potential of the plant for element accumulation and greater transfer capacity of an element from root to shoot, respectively^27^. In the present study, over the range of 50–200 mg/L of B supply, the BCF values in the shoot of *I*. *wilsonii* (41.30–26.47) and *P*. *tenuiflora* (56.31–26.44) were much greater than those of *S. glauca* (18.77–8.78) (Table 1). These results demonstrate that *I*. *wilsonii* and *P*. *tenuiflora* have a greater accumulation capacity than *S. glauca*. Along with high BCF values, high TF values were also observed in *I*. *wilsonii*, indicating that, at extremely high B concentrations, *I*. *wilsonii* did not restrict B uptake and its translocation from root to shoot. According to previous studies, for the same plant species, the tolerant varieties usually accumulate less B in shoot than sensitive varieties^31^. However, this rule may not be suitable for the comparison between different species. Under high B conditions, *I*. *wilsonii* seems to have a different mechanism of B tolerance, which remains to be determined. Nevertheless, these findings have provided evidence that *I*. *wilsonii* will be a most promising candidate for the phytoremediation of high-B soil. Although *I*. *wilsonii* has been used for removing nitrogen and phosphorus from water as candidate plant of constructed wetland^36^, the removal of B by *I*. *wilsonii* has not been investigated in constructed wetlands. In our future research, *I*. *wilsonii* will be applied to the treatment of high-B saline water using constructed wetland systems. Plants have been proved to play a direct role in B removal process in constructed wetlands^37,38^. Therefore, *I*. *wilsonii* might be a promising candidate for constructed wetlands in saline areas.

Many species of *Puccinellia*, such as *P*. *distans* and *P*. *frigida*, are known for their extremely high tolerance to B, and they also have been widely considered as potential candidates for the phytoremediation of B-contaminated sites^6,30,34,39,40^. In the present work, *P*. *tenuiflora* also has been proved to be tolerant to B and capable of B accumulation, confirming that *Puccinellia* is a B-tolerant genus. The other three plants, *S. glauca*, *I*. *wilsonii*, and *P*. *tenuiflora*, have not yet been studied for B tolerance and B enrichment. Since many species of *Puccinellia* are also tolerant to salt, more species of this genus should be screened for the remediation of soils with high salinities. In our laboratory, we have confirmed that the other three plants *S. glauca*, *I*. *wilsonii*, and *P*. *tenuiflora* are also more salt-tolerant species. Although most species of *Puccinellia* have small biomass, which is a disadvantage for B accumulation, their high growth rate may allow them as an initial cover for the restoration of B-contaminated soil.

The four species we used are all seedlings which are more sensitive to B than adult individuals. Although two weeks test period is not so long, it is enough to evaluate B toxicity in plant and to screen the species with high B tolerance and accumulation. It is important to note that our data are obtained under room conditions with plants cultivated in hydroponic systems. Therefore, it is hard to identify these three species as hyperaccumulators of B. But we believe that the present study will provide the scientific theoretical basis for controlling B-contaminated soils and water by phytoremediation technology in reality. Absolutely, more research and further verification in practical applications were required. In our future studies, plant growth rate will be determined to know whether the candidate plant species can absorb B within a reasonable time and whether the accumulation is decreased by extreme growth reduction^34^. In addition, because B toxicity and salt stress often occur simultaneously, the interaction of B and salt should be considered. Despite the four test species are all salt-tolerant, it should be emphasized that they should be evaluated under the combined stresses of B and salt before being applied in the phytoremediation of B-contaminated soils.

## Conclusions

Among the four tested plant species, *I*. *wilsonii* is the most tolerant species to B toxicity and has the highest B accumulation capacity. *S. glauca* and *P*. *tenuiflora* show similar B tolerance, but *P*. *tenuiflora* has a greater B accumulation capacity than *S. glauca*. *T*. *pannonicum* has the lowest tolerance to B and the lowest accumulation capacity. Our results suggest that *S. glauca*, *I*. *wilsonii*, and *P*. *tenuiflora* are suitable for the phytoremediation of high-B soil with high salinities. We propose that *I*. *wilsonii* as a most promising candidate for B-phytoremediation because of its great capacity for B tolerance and accumulation.
